# Association of FAS gene polymorphisms with systemic lupus erythematosus: A case-control study and meta-analysis

**DOI:** 10.3892/etm.2012.625

**Published:** 2012-06-28

**Authors:** MAN-MAN LU, QIAN-LING YE, CHEN-CHEN FENG, JIE YANG, TAO ZHANG, JING LI, RUI-XUE LENG, HAI-FENG PAN, HUI YUAN, DONG-QING YE

**Affiliations:** 1 Department of Epidemiology and Biostatistics, School of Public Health, and; 2 Anhui Provincial Laboratory of Population Health and Major Disease Screening and Diagnosis, Anhui Medical University, Hefei, Anhui 230032;; 3 The Second Affiliated Hospital of Anhui Medical University, Hefei, Anhui 230601;; 4 Anhui Center for Disease Control and Prevention, Hefei, Anhui 230601;; 5 Department of Preventive Medicine, Wannan Medical College, Wuhu, Anhui 241002, P.R. China

**Keywords:** systemic lupus erythematosus, FAS, single-nucleotide polymorphisms, meta-analysis

## Abstract

The association of functional polymorphisms in the promoter of the apoptosis gene FAS with systemic lupus erythematosus (SLE) susceptibility has been a controversial subject. We conducted a case-control study to investigate this association in a Chinese population and performed a meta-analysis in different populations. The single nucleotide polymorphisms (SNPs) rs2234767 (−1377G>A) and rs1800682 (−670A>G) were genotyped by TaqMan allelic discrimination assays in 552 Chinese SLE patients and 718 healthy controls. In our case-control study, we observed allelic association between the promoter SNP rs2234767 [P=0.033, odds ratio (OR)=0.836, 95% confidence interval (CI), 0.709–0.986] and SLE but not the SNP rs1800682. Haplotype analysis revealed that one haplotype of GA was significantly associated with the disease (P=0.039, OR=1.184, 95% CI, 1.009–1.391). In the meta-analysis available studies, including our data, were combined using the STATA software package v.7.0. The meta-analysis revealed a significant association between FAS polymorphisms and SLE (rs2234767 A vs. G allele; P=0.004, OR=0.819, 95% CI, 0.715–0.938, rs1800682 G vs. A allele: P=0.034, OR=0.791, 95% CI, 0.637–0.983). In conclusion, FAS gene polymorphisms may contribute to SLE susceptibility in the Chinese population, and the meta-analysis shows that FAS polymorphisms may be associated with SLE susceptibility in different populations.

## Introduction

Systemic lupus erythematosus (SLE) is a prototypic auto-immune disease, characterized by autoantibody production, immune complex formation and multiple organ damage (1). Although the pathogenic mechanisms of SLE are not yet fully understood, previous studies have indicated that abnormalities of apoptosis may be involved in the development of autoimmune disorders (2,3). SLE patients demonstrated accelerated apoptosis of circulating lymphocytes and delayed clearance of apoptotic cells (4). The excess of lymphocyte apoptosis and deficient clearance of apoptotic cells may contribute to B-cell hyperactivity and subsequent autoantibody overproduction (5,6). Accordingly, FAS, as the major mediator of the induction of apoptosis in activated lymphocytes, has received more attention in SLE.

FAS (CD95/APO-1/TNFSF6), as a transmembrane receptor among cell surface death receptors, belongs to the tumor necrosis factor receptor (TNFR) superfamily. It is expressed on numerous types of immune cells (7) and plays a key role in the homeostasis of immune cells, regulation of T lymphocytes, and elimination of infected and malignant cells. When the FAS receptor is cross-linked, either by its natural ligand (FASL) or by specific monoclonal antibodies, the target cell undergoes apoptosis (8). Enhanced or defective FAS-mediated apoptosis may result in an impaired clearance of apoptotic cells or failure to eliminate autoreactive cells (9), which is one of the susceptibility factors of SLE development.

The human FAS gene, which is mapped to chromosome 10q24.1, consists of nine exons and eight introns (10). Previously, two single nucleotide polymorphisms (SNPs) (−1377G>A, rs2234767 and −670A>G, rs1800682) located in the promoter region of the FAS gene have been examined for relevance in a number of autoimmune diseases including SLE (11–16). These findings were not always consistent and a number of issues are controversial. Until now, little is known with regard to the correlation between SNPs in the FAS gene and SLE susceptibility in the Chinese population. In the present study, we investigated the association between the two SNPs in the FAS promoter and SLE susceptibility in the Chinese population. We also performed a meta-analysis on all eligible published case-control studies including the present study, to assess the association.

## Materials and methods

### Case-control study

#### Patients and healthy controls

A total of 552 patients with SLE were recruited from Anhui Provincial Hospital and The First Affiliated Hospital of Anhui Medical University. All patients fulfilled the 1997 revised criteria of the American College of Rheumatology for the classification of SLE (17). Their mean age was 37.53±12.34 years. A total of 718 healthy blood donors were included as controls, all of whom did not have SLE or other autoimmune diseases. Their mean age was 36.54±16.70 years. All patients and controls were ethnic Han Chinese. Patient written consent was provided in order to participate.

#### DNA samples and genotyping

EDTA anti-coagulated venous blood samples were obtained from all the participants. Genomic DNA was extracted from peripheral blood lymphocytes by standard procedures using Flexi Gene DNA kits (Qiagen, Hilden, Germany). All samples were stored at −80°C prior to testing. A total of two SNPs were genotyped by the TaqMan SNP Genotyping Assay kit (Applied Biosystems, Foster City, CA, USA; catalogue nos. C_12123966_10 for rs2234767, C_9578811_10 for rs1800682). Real-time polymerase chain reaction (PCR) was performed on the ABI PRISM 7300 (Applied Biosystems, Foster City, CA, USA). The reaction conditions were initially denatured at 95°C for 10 min followed by 45 cycles of denaturing at 95°C for 15 sec and annealing/extension at 60°C for 1 min.

#### Statistical analysis

Data analysis was performed using SPSS 10.0 software (SPSS Inc.; 2000). The Chi-square test or Fisher's exact test was used to estimate the Hardy-Weinberg equilibrium (HWE) in both SLE patients and healthy controls, as well as to compare the genotype and allele frequencies between the two groups. Odds ratios (ORs) and 95% confidence intervals (CIs) were also calculated. The multiple-locus haplotypes comprising the two SNPs were estimated by comparing the difference in the haplotype frequencies for the overall subjects with the SHEsis software (18). In the two-tailed test a probability level of <0.05 was considered to indicate a statistically significant result. The power analysis was performed using the statistical program G^*^Power (http://www.psycho.uniduesseldorf.de/aap/projects/gpower).

### Meta-analysis

#### Identification of eligible studies

All studies examining the association between FAS polymorphism and SLE were fully considered and carefully selected. Three electronic databases (PubMed, Embase and Web of Science) were searched using the search terms ‘FAS’, ‘TNFSF6’, ‘CD95’, ‘APO-1’, ‘systemic lupus erythematosus’ and ‘SLE’. The most recent search was updated in March 2012. We only recruited data from fully published papers, not meeting or conference abstracts. The inclusion criteria were as follows; i) independent case-control design, ii) available allele frequency or genotype distribution data and iii) sufficient published data for estimating OR with 95% CI.

#### Meta-analysis methods

Meta-analysis was performed using the STATA software package v.7.0 (Stata Corporation, College Station, TX, USA). Cochran's Q-statistic and inconsistency (I^2^) values were presented to assess the heterogeneity (19). When a significant statistic (P<0.10 or I^2^>50%) indicated heterogeneity across studies, the random effects model was used, and when heterogeneity was not indicated across studies, the fixed effects model was used. The Z test was used to determine the significance of overall effect (20,21). Begg's funnel plot and Egger's test were performed to analyze the publication bias (22). P<0.05 was considered to indicate a statistically significant result.

## Results

### Case-control study

#### Association between FAS polymorphisms and SLE

The allelic and genotypic frequencies of two SNPs in the FAS gene in patients and controls are listed in Table I. Case-control comparison revealed a significant association between SLE and the minor allele A at SNP rs2234767 (P=0.033, OR=0.836, 95% CI, 0.709–0.986). Significant differences in genotypic distribution were also observed in SLE patients and controls (AG vs. GG, P=0.041, OR=0.779, 95% CI, 0.613–0.990; AG+AA vs. GG, P=0.024, OR=0.773, 95% CI, 0.618–0.967). However, no significant differences were demonstrated in the allelic and genotypic distributions at SNP rs1800682 between patients and controls.

#### Haplotype analysis

The association between the frequencies of haplotypes and SLE was also estimated. A total of four (22) possible haplotypes (AG, GA, GG and AA) were constructed from two SNPs. Analysis of the haplotypes revealed that the haplotype GA was significantly associated with SLE (P=0.039, OR=1.184, 95% CI, 1.009–1.391; Table II). Thus, the GA haplotype appeared to indicate an increased risk of SLE. The HWE P-value in controls was >0.05. Strong linkage disequilibrium (LD) was observed between the two SNPs (D'=0.937, r^2^=0.708). We estimated that our study has a statistical power of 94.6% by the statistical program G^*^Power.

### Meta-analysis

#### Studies included in the meta-analysis

In the meta-analysis, four studies (12,13,16) were identified with regard to the SNP rs2234767 (three published articles and the present study). These studies were included and up to 996 patients with SLE and 1160 controls were combined. A total of six studies (11–15) were identified with regard to the SNP rs1800682 (five published articles and one current study). In total 1,122 patients with SLE and 1,339 controls were recruited in this meta-analysis. The pooled population originated from Asia and Europe.

#### Meta-analysis of the FAS polymorphisms and SLE susceptibility

**Figure 1:**
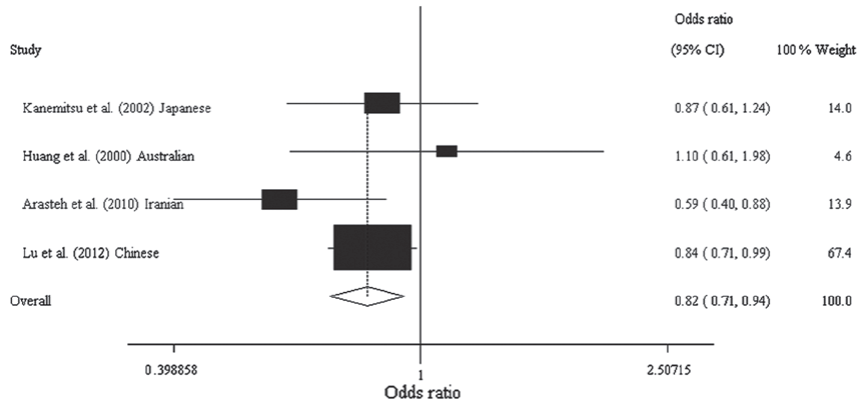
Forest plot for the meta-analysis of the association between the FAS rs2234767 polymorphism and SLE (A vs. G). CI, confidence interval; SLE, systemic lupus erythematosus.

**Figure 2:**
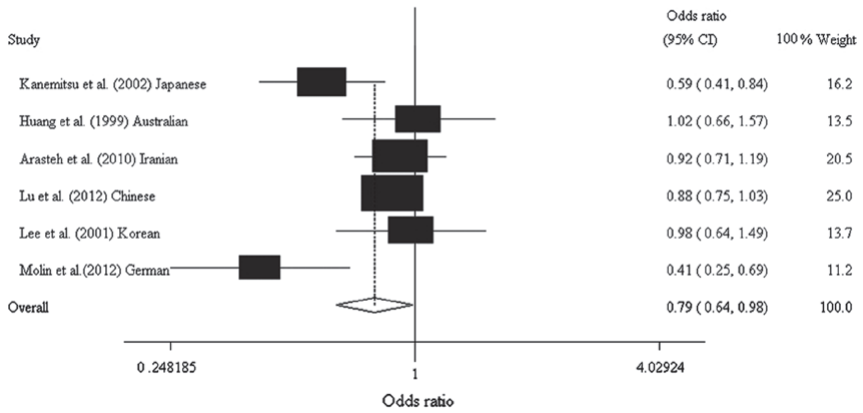
Forest plot for the meta-analysis of the association between the FAS rs1800682 polymorphism and SLE (G vs. A). CI, confidence interval; SLE, systemic lupus erythematosus.

A summary of the meta-analysis findings on the association between the FAS polymorphisms and SLE is provided in Table III. The meta-analysis showed that the two SNPs were significantly associated with SLE susceptibility rs2234767 A vs. G allele; P=0.004, OR=0.819, 95% CI, 0.715–0.938; rs1800682 G vs. A allele; P=0.034, OR=0.791, 95% CI, 0.637–0.983; Table IV, Figs. 1 and 2). Analysis following stratification by population indicated that the two SNPs were significantly associated with SLE in Asian populations (rs2234767 A vs. G allele; P=0.002, OR=0.805, 95% CI, 0.700–0.926; rs1800682 G vs. A allele; P=0.012, OR=0.855, 95% CI, 0.758–0.966). The meta-analysis also showed that the dominant effects of rs2234767 and rs1800682 were associated with the susceptibility to SLE in overall (AG+AA vs. GG; P=0.002, OR=0.751, 95% CI, 0.628–0.898; AG+GG vs. AA; P=0.015, OR=0.646, 95% CI, 0.454–0.920, respectively) and Asian populations (AG+AA vs. GG; P=0.001, OR=0.730, 95% CI, 0.606–0.880; AG+GG vs. AA; P=0.034, OR=0.711, 95% CI, 0.518–0.975, respectively; Table IV).

#### Evaluation of study quality and heterogeneity

The distribution of rs2234767 and rs1800682 genotypes in control groups was consistent with the HWE in all studies (Table III). We evaluated that the meta-analysis had statistical powers of 99.6 and 99.9% for rs2234767 and rs1800682, respectively, using the program G^*^Power. Heterogeneity was demonstrated in the meta-analysis for the rs1800682 but not for the rs2234767 polymorphism. Egger's regression test showed no evidence of publication bias in this meta-analysis of FAS polymorphisms in any of the studies included (Egger's regression test P>0.1; Table IV).

## Discussion

Evidence has accumulated that FAS-mediated apoptosis may be involved in the pathogenesis of SLE. The expression of FAS on peripheral blood lymphocytes has been reported to be upregulated in patients with SLE (23). The variation of the FAS gene in the promoter region was described to be associated with the differential expression of the gene in SLE (24). The SNPs rs2234767 and rs1800682 are located in the promoter of the FAS gene; the two SNPs have been shown to interfere with the SP1 and STAT1 transcription factor binding sites, respectively, affecting promoter activity and in turn FAS gene expression (24,25). Thus, based on the function of the two SNPs, variation may have an effect on susceptibility to SLE.

In our case-control study, we analyzed the frequencies of alleles and genotypes at the two SNPs in 552 SLE patients and 718 healthy controls of the Chinese population. We showed that the A allele frequency of rs2234767 is associated with SLE susceptibility. Significant differences in the genotype frequencies of AG vs. GG, AG+AA vs. GG at rs2234767 were observed. However, on evaluation of allele and genotype distributions at rs1800682, no significant differences were observed between patients and controls. Analysis of the haplotypes revealed that individuals carrying the haplotype GA had an increased risk of SLE. The results of the haplotype analysis were concordant with the suggestion that the rs2234767 G allele was mainly associated with the rs1800682 A allele in SLE patients by Kanemitsu *et al* (13). They observed that the rs1800682 A allele with higher STAT1 binding activity may result in the alteration of FAS gene expression. Previous studies have suggested that STAT1 binding alone cannot be equated with a biological function and that concomitant SP1 binding may influence transcriptional activation (26). This complex association may explain why the GA haplotype shows an increased risk of SLE.

The repeated investigation of the association of FAS polymorphisms with SLE in different populations supports the involvement of this gene in SLE susceptibility. However, certain conclusions are inconsistent. For example, studies by Molin *et al* (11) and Kanemitsu *et al* (13) demonstrated an association between rs1800682 and SLE, however, the association was not identified in Iranian (12), Korean (14), Australian (15) populations and, in our present study, in a Chinese population. In a study by Arasteh *et al* (12), the SNP rs2234767 showed a significant association with SLE, but this was not observed in Japanese (13) and Australian populations (16). Due to the relatively small samples in these studies, the results were not convincing and needed to be refined. Thereby we performed a meta-analysis with all available studies (published and our present results) to investigate the association between the FAS polymorphisms and SLE. The result of our meta-analysis demonstrated the association of the two SNPs with SLE susceptibility. Analysis following stratification by population detected a significant association with the FAS polymorphisms in Asian individuals. The meta-analysis revealed a dominant effect associated with susceptibility to SLE at the two SNPs overall and in Asian populations.

The positive result of rs2234767 was presented by our replication study and confirmed by meta-analysis. Although our study did not find an association between rs1800682 and SLE in the Chinese population, the association was identified in overall and Asian populations in the meta-analysis. Heterogeneity was observed in the meta-analysis for rs1800682 and SLE. One of the reasons for this disparity may be due to population differences in genotype distributions. In addition, we observed a strong LD between the two SNPs in our study population, but a weaker LD was suggested in the Japanese population (13). Therefore the inconsistent genetic association with the two SNPs in different populations, including the present study may be due to different LD structure. In addition, SLE is a multi-factorial disease; individual exposure to various environmental factors in combination with genetic susceptibility may have contributed to the conflicting results.

There are certain limitations of this meta-analysis that should be discussed. First, the number of studies and the number of subjects in the studies included in the meta-analysis by the disease were small. Second, when we explored the association between rs1800682 and SLE, heterogeneity was observed. Third, our ethnicity-specific meta-analysis included data from individuals with Asian and European origin, and thus the results are applicable to only these groups. In the subgroup analysis, the majority of the studies were performed in populations of Asian descent. Further studies are therefore required in other ethnic populations. Despite these limitations, our meta-analysis confirmed an association between SLE and FAS gene polymorphisms.

Our investigation provided evidence that FAS gene polymorphisms contributed to SLE susceptibility in the Chinese population. The combined results of independent association studies by meta-analysis showed significant association between FAS polymorphisms and SLE. The investigation of the genetic basis of SLE in other populations may advance the overall understanding of the pathogenesis of this disease. Further studies are still required in larger numbers of samples and other ethnic populations.

## Figures and Tables

**Table I:** Association between FAS polymorphisms and SLE.

Polymorphism	SLE patients n (%)	Controls n (%)	P-value	OR (95% CI)
rs2234767				
Allele				
G	737 (66.8)	900 (62.7)		-
A	367 (33.2)	536 (37.3)	0.033	0.836 (0.709–0.986)
Genotype				
GG	257 (46.6)	289 (40.3)		-
AG	223 (40.4)	322 (44.8)	0.041	0.779 (0.613–0.990)
AA	72 (13.0)	107 (14.9)	0.110	0.757 (0.537–1.066)
AG+AA	295 (53.4)	429 (59.7)	0.024	0.773 (0.618–0.967)
rs1800682				
Allele				
A	675 (61.1)	834 (58.1)		-
G	429 (38.9)	602 (41.9)	0.119	0.880 (0.750–1.033)
Genotype				
AA	219 (39.7)	254 (35.4)		-
AG	237 (42.9)	326 (45.4)	0.175	0.843 (0.659–1.079)
GG	96 (17.4)	138 (19.2)	0.184	0.807 (0.588–1.108)
AG+GG	333 (60.3)	464 (64.6)	0.116	0.832 (0.662–1.047)

SLE, systemic lupus erythematosus; OR, odds ratio; CI, confidence interval.

**Table II:** Haplotype analysis of FAS polymorphisms in SLE patients and controls.

Haplotype	SLE patients n (%)	Controls n (%)	χ^2^	P-value	OR (95% CI)
rs2234767-rs1800682					
AG	367 (33.2)	502 (34.9)	1.771	0.183	0.893 (0.757–1.055)
GA	675 (61.1)	800 (55.7)	4.266	0.039	1.184 (1.009–1.391)
GG	62 (5.6)	100 (7.0)	2.421	0.120	0.772 (0.557–1.070)
AA	0 (0.0)	34 (2.4)	-	-	-
Global	1104	1436	5.176	0.075	-

SLE, systemic lupus erythematosus; OR, odds ratio; CI, confidence interval.

**Table III:** Characteristics of the individual studies included in the meta-analysis.

			Patients (n)		MAF			
Study (Ref.)	Nationality	Ethnicity	SLE	Control	SLE	Control	HWE	OR (95% CI)
rs2234767								
Kanemitsu *et al* (13)	Japanese	Asian	109	140	0.420	0.460	0.202	0.867 (0.607–1.239)
Huang *et al* (16)	Australian	European origin	86	90	0.160	0.130	0.917	1.103 (0.615–1.978)
Arasteh *et al* (12)	Iranian	Asian	249	212	0.098	0.156	0.652	0.592 (0.399–0.879)
Lu *et al* (Present study)	Chinese	Asian	552	718	0.332	0.373	0.266	0.836 (0.709–0.986)
Total			996	1160	0.269	0.326		0.819 (0.715–0.938)
rs1800682								
Kanemitsu *et al* (13)	Japanese	Asian	109	140	0.450	0.590	0.094	0.588 (0.412–0.841)
Huang *et al* (15)	Australian	European origin	79	86	0.490	0.490	0.825	1.021 (0.663–1.573)
Arasteh *et al* (12)	Iranian	Asian	249	212	0.484	0.505	0.272	0.920 (0.710–1.192)
Lu *et al* (Present study)	Chinese	Asian	552	718	0.389	0.419	0.070	0.880 (0.750–1.033)
Lee *et al* (14)	Korean	Asian	87	87	0.420	0.430	0.230	0.977 (0.638–1.495)
Molin *et al* (11)	German	European origin	46	96	0.402	0.620	0.210	0.413 (0.248–0.686)
Total			1122	1339	0.466	0.509		0.791 (0.637–0.983)

SLE, systemic lupus erythematosus; OR, odds ratio; CI, confidence interval; MAF, minor allele frequency; HWE, Hardy-Weinberg equilibrium.

**Table IV:** Meta-analysis of the association between FAS polymorphisms and SLE.

			Test of association			Test of heterogeneity			
SNPs	Population	No. of studies	OR	95% CI	P-value	I^2^	P-value	Model	Publication bias P-value
rs2234767									
A vs. G allele	Overall	4	0.819	0.715–0.938	0.004	20.1	0.289	F	0.969
	Asian	3	0.805	0.700–0.926	0.002	25.9	0.259	F	0.586
	European origin	1	1.103	0.615–1.978	0.742	NA	NA	NA	NA
AG+AA vs. GG (Dominant)	Overall	4	0.751	0.628–0.898	0.002	0.0	0.462	F	0.917
	Asian	3	0.730	0.606–0.880	0.001	0.0	0.490	F	0.587
	European origin	1	1.065	0.548–2.067	0.853	NA	NA	NA	NA
AA vs. GG+AG (Recessive)	Overall	4	0.865	0.659–1.137	0.300	0.0	0.659	F	0.940
	Asian	3	0.853	0.647–1.125	0.260	0.0	0.559	F	0.536
	European origin	1	1.590	0.259–9.758	0.616	NA	NA	NA	NA
rs1800682									
G vs. A allele	Overall	6	0.791	0.637–0.983	0.034	62.6	0.020	R	0.384
	Asian	4	0.855	0.758–0.966	0.012	40.2	0.171	F	0.670
	European origin	2	0.656	0.270–1.594	0.352	85.9	0.008	R	NA
AG+GG vs. AA (Dominant)	Overall	6	0.646	0.454–0.920	0.015	65.6	0.013	R	0.256
	Asian	4	0.711	0.518–0.975	0.034	53.9	0.089	R	0.489
	European origin	2	0.469	0.112–1.958	0.299	84.9	0.010	R	NA
GG vs. AA+AG (Recessive)	Overall	6	0.889	0.732–1.079	0.234	15.6	0.314	F	0.512
	Asian	4	0.921	0.747–1.134	0.438	0.0	0.608	F	0.970
	European origin	2	0.675	0.237–1.926	0.463	71.3	0.062	R	NA

SLE, systemic lupus erythematosus; OR, odds ratio; CI, confidence interval; SNPs, single nucleotide polymorphisms; F, fixed model; R, random model; NA, not available.
